# Maternal Retroperitoneal Cystic Lymphangioma During Pregnancy

**DOI:** 10.7759/cureus.99639

**Published:** 2025-12-19

**Authors:** Marta Campos, Marta Xavier, Mónica Melo, Susana Graça, Claudina Carvalho

**Affiliations:** 1 Department of Obstetrics and Gynecology, Gaia/Espinho Local Health Unit, Vila Nova de Gaia, PRT; 2 Department of Surgery, Gaia/Espinho Local Health Unit, Vila Nova de Gaia, PRT

**Keywords:** cystic lymphangioma, lymphangioma, percutaneous drainage, pregnancy, retroperitoneum

## Abstract

Retroperitoneal cystic lymphangiomas are rare benign tumors, and their occurrence during pregnancy is exceptionally uncommon. We describe a case of a 31-year-old patient at 36 weeks of pregnancy with a large retroperitoneal tumor discovered incidentally during routine obstetric ultrasound (US). One week later, at 37 weeks of gestation, she delivered a healthy newborn via cesarean section. Although surgical excision was initially proposed, the lesion showed spontaneous dimensional reduction in the postpartum period. Given this atypical evolution, percutaneous drainage followed by sclerosis was performed. To our knowledge, this is only the second reported case of retroperitoneal cystic lymphangioma diagnosed during pregnancy successfully managed with a less invasive approach than complete surgical resection, highlighting the potential of conservative management in selected cases.

## Introduction

Lymphangiomas are extremely rare benign malformations of the lymphatic system, resulting from failure of lymphatic vessel development and drainage, and are characterized by dilated lymphatic channels lined by endothelial cells [1-4]. They are most commonly found in childhood and are rare in adults [3,5-7]. These tumors can occur in any part of the body, being more frequent in extra-abdominal locations such as the head, neck, and axilla. Only approximately 1% of lymphangiomas are located in the retroperitoneum [1-4,7].

Retroperitoneal lymphangiomas may remain asymptomatic for long periods and are frequently discovered incidentally during imaging studies performed for other reasons [2,7-9]. When symptoms occur, they are typically nonspecific and may include abdominal or lumbar pain, weight loss, or gastrointestinal complaints [1,9]. Imaging modalities such as ultrasound (US), computed tomography (CT), and magnetic resonance imaging (MRI) are fundamental for characterizing the lesion and planning the therapeutic approach [1,4,5,7,8]. MRI is particularly advantageous during pregnancy because it allows multiplanar evaluation without ionizing radiation [1,4].

Complete surgical excision is the treatment of choice, both to confirm the diagnosis histologically and to prevent recurrence [3-5,7-9]. However, during pregnancy, management should be individualized based on symptoms, lesion size, and risk of complications [4].

The presence of lymphangiomas during pregnancy is uncommon, with only 11 cases reported in the literature and just one with retroperitoneal location [4,8]. This case is distinctive not only for its occurrence during pregnancy, but also because spontaneous regression after delivery enabled a successful minimally invasive treatment instead of the typically recommended complete surgical excision.

## Case presentation

**Figure 1:**
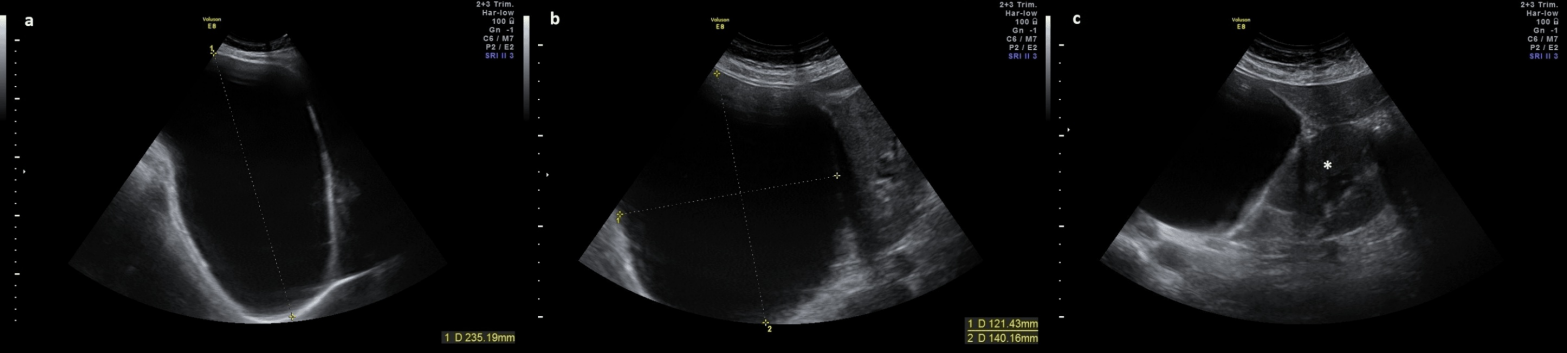
Antenatal abdominal US at 36 weeks of gestation showing a large, elongated anechoic retrohepatic cystic lesion with regular contours, measuring 235 × 140 × 121 mm, causing inferior and medial deviation of the right kidney. No Doppler flow was observed. Due to the deep retroperitoneal location, the uterus and the lesion cannot be visualized in the same image. (a) Coronal section. (b) Axial section. (c) Axial section showing the cystic lesion and right kidney (*). US: ultrasound

A 31-year-old woman, primigravida, at 36 weeks of gestation, was referred to our center for hospital prenatal registration appointment. Her personal and family medical history was irrelevant, and pregnancy was uneventful. During the appointment, an obstetric US showed late fetal growth restriction (estimated fetal weight < third centile) and incidentally identified a maternal retrohepatic cystic lesion measuring 235 x 140 x 121 mm, causing inferior and medial displacement of the right kidney (Figure 1).

**Figure 2:**
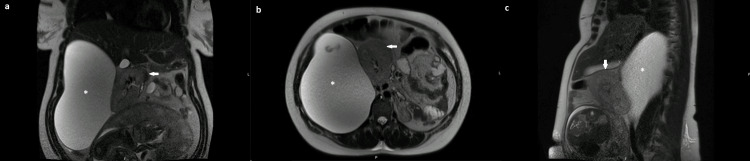
Abdominal MRI: T2 sequence: very large retroperitoneal lesion in the right flank (*), with homogeneous hypersignal pattern and regular and well-defined contours, pushing the bowel loops and the right kidney anteriorly, molding to the posterior surface of the right kidney. (a) Coronal section. (b) Axial section. (c) Right parasagittal section. The arrow points to the right kidney with medial and anterior displacement. MRI: magnetic resonance imaging

The patient was asymptomatic, and both physical and abdominal examinations were normal. Tumor markers revealed an elevated alpha-fetoprotein (AFP) level of 74.10 ng/mL and negative carcinoembryonic antigen (CEA), carbohydrate antigen 125 (CA 125), and carbohydrate antigen 19-9 (CA 19-9) levels. Non-contrast MRI showed a very large lesion in the right flank measuring 145 x 170 x 220 mm, with regular and well-defined contours, pushing the bowel loops and the right kidney anteriorly, suggesting a retroperitoneal origin, molding to the posterior surface of the right kidney (Figure 2). Due to its large size, it was not possible to establish the etiology of the lesion.

A multidisciplinary meeting was held involving maternal and fetal medicine and general surgery, concluding that the lesion was probably compatible with a retroperitoneal cystic lymphangioma. Due to the risk of cyst rupture during Valsalva maneuvers, a cesarean section was performed at 37 weeks of gestation, and a healthy male newborn was delivered with a birthweight of 1,910 g (< first centile) and Apgar scores of 9, 10, 10 at 1, 5, and 10 minutes, respectively. Excision of the lesion during the cesarean section was not attempted due to its size, deep retroperitoneal position, and close proximity to major vascular and renal structures, which posed a significant risk of intraoperative complications. The postoperative period was uneventful, with uncomplicated puerperium. Histological examination of the placenta shows changes consistent with maternal perfusion impairment.

**Figure 3:**
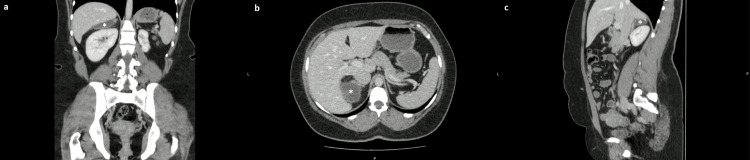
Abdominal CT performed after the percutaneous drainage and sclerosis of the cystic lesion: cystic lesion (*) with somewhat lobulated contours, without solid components adjacent to the upper pole of the right kidney, the right adrenal gland, and the lower surface of the right hepatic lobe, measuring 58 × 34 × 14 mm. (a) Coronal section. (b) Axial section. (c) Right parasagittal section. CT: computed tomography

A follow-up MRI performed four months after delivery showed no significant change in lesion size. The patient remained asymptomatic. Nevertheless, surgical excision was proposed, as it is the recommended first-line treatment. However, on the preoperative US, eight months after the MRI, a marked spontaneous reduction in the lesion’s size was observed (largest diameter approximately 10 cm). Given the extensive dissection required with the consequent high surgical risk and the significant reduction in lesion size, percutaneous drainage and sclerosis was chosen. Under imaging guidance, cystic fluid was aspirated, and the aspirated fluid was not sent for laboratory analysis, as decided by the interventional radiology team. Subsequently, 96% ethanol was instilled as the sclerosant agent. The procedure was uncomplicated, and no immediate minor or major adverse reactions were observed. Four months after the procedure, the patient was asymptomatic, and a CT scan showed a significantly smaller cystic lesion adjacent to the upper pole of the right kidney, the right adrenal gland, and the inferior surface of the right hepatic lobe, measuring 58 x 34 x 14 mm (Figure 3). At the most recent follow-up, a little over three years after the procedure, the patient remained asymptomatic, and CT imaging demonstrated continued regression of the lesion with no evidence of recurrence.

## Discussion

Most retroperitoneal lymphangiomas are asymptomatic and are discovered incidentally during imaging examinations performed for other reasons [2,7-9]. Signs and symptoms, when present, can be quite variable, with the most common being abdominal or lower back pain, weight loss, fatigue, fever, or hematuria [1,9]. In the presented case, the patient was asymptomatic, with no abnormalities found on physical or abdominal examination. The lesion was detected incidentally during an obstetric US. Multiple imaging methods, such as US, CT, and MRI, can be used for the diagnosis of cystic lymphangiomas, allowing the description of the lesion, size, and relationship with adjacent structures [1,4,5,7,8]. In pregnancy, MRI is the preferred method to evaluate retroperitoneal tumors because it provides images in multiple planes without loss of resolution, allowing the characterization of the lesions and assessing surgical resectability [1,4]. The definitive diagnosis requires histological evaluation after surgical resection [3-5,7-9]. The role of tumor markers in this type of lesion is unknown [4]. In our case, an elevation of AFP was observed, a finding that must be interpreted with caution, as AFP physiologically increases during the third trimester of pregnancy and therefore lacks specificity for malignancy [4].

The recommended treatment for retroperitoneal cystic lymphangioma is surgical resection [3-5,7-9]. During pregnancy, the therapeutic approach should be personalized, based on the symptoms and tumor location [4]. Given its benign nature, expectant management is recommended, with clinical, imaging, and fetal well-being assessments to monitor for lesion growth or the development of complications [4]. In the absence of complications, surgical resection is recommended after delivery [4]. In the presented case, considering that the pregnant woman was asymptomatic and there were no complications associated with the lesion, a multidisciplinary meeting decided on surgical resection after delivery. There is no recommended delivery route based on the diagnosis of lymphangioma [4]. In our case, considering the size of the lesion, a cesarean section was chosen to reduce potential complications associated with expulsive efforts. Excision was not attempted during the cesarean section due to the lesion’s deep retroperitoneal location and proximity to vital structures, which would have significantly increased operative risk. Besides surgical resection, aspiration of the lesion’s contents, with or without injection of sclerosant agents, is another therapeutic option that has proven effective in selected cases [2,5,9]. However, it is associated with higher infection and recurrence rates [2,5,9]. In this patient, a substantial spontaneous reduction in lesion size was observed on the preoperative US. This unexpected evolution was the key factor enabling the choice of a minimally invasive treatment approach rather than complete surgical removal. Given the benign nature of the lesion, the lack of symptoms, and the decreasing size, percutaneous drainage followed by sclerosis was deemed an appropriate and safe alternative. The patient remains asymptomatic and is being monitored with annual follow-up imaging, given the known risk of recurrence.

## Conclusions

In conclusion, this case illustrates a rare presentation of retroperitoneal cystic lymphangioma diagnosed during pregnancy and emphasizes the importance of individualized management. In an asymptomatic patient with spontaneous reduction in lesion size, a conservative approach offered a safe and effective alternative to surgical excision. Image-guided percutaneous drainage and sclerosis allowed resolution of the mass while avoiding the risks associated with extensive retroperitoneal surgery. Close follow-up remains essential due to the possibility of recurrence. This case reinforces that less invasive management strategies may be appropriate in carefully selected patients. Therefore, the main limitation of this case is the absence of a histological diagnosis. However, given the extraordinary advancements in imaging techniques, the diagnosis can be assumed with reassuring certainty.
